# Integrative Analysis Identified Key Schizophrenia Risk Factors from an Abnormal Behavior Mouse Gene Set

**DOI:** 10.3390/life11020172

**Published:** 2021-02-23

**Authors:** Miao Chen, Weidi Wang, Weicheng Song, Wei Qian, Guan Ning Lin

**Affiliations:** 1 School of Biomedical Engineering, Shanghai Jiao Tong University, Shanghai 200030, China; chenmiao95@sjtu.edu.cn (M.C.); wwd@smhc.org.cn (W.W.); goubegou@sjtu.edu.cn (W.S.); kentergav@sjtu.edu.cn (W.Q.); 2 Engineering Research Center of Digital Medicine and Clinical Translational, Ministry of Education of China, Shanghai 200030, China

**Keywords:** schizophrenia, abnormal behavior gene set, region, differentially expressed genes, *de novo* mutation, copy number variant

## Abstract

Schizophrenia (SCZ) is a severe chronic psychiatric illness with heterogeneous symptoms. However, the pathogenesis of SCZ is unclear, and the number of well-defined SCZ risk factors is limited. We hypothesized that an abnormal behavior (AB) gene set verified by mouse model experiments can be used to better understand SCZ risks. In this work, we carried out an integrative bioinformatics analysis to study two types of risk genes that are either differentially expressed (DEGs) in the case-control study data or carry reported SCZ genetic variants (MUTs). Next, we used RNA-Seq expression data from the hippocampus (HIPPO) and dorsolateral prefrontal cortex (DLPFC) to define the key genes affected by different types (DEGs and MUTs) in different brain regions (DLPFC and HIPPO): DLPFC-kDEG, DLPFC-kMUT, HIPPO-kDEG, and HIPPO-kMUT. The four hub genes (SHANK1, SHANK2, DLG4, and NLGN3) of the biological functionally enriched terms were strongly linked to SCZ via gene co-expression network analysis. Then, we observed that specific spatial expressions of DLPFC-kMUT and HIPPO-kMUT were convergent in the early stages and divergent in the later stages of development. In addition, all four types of key genes showed significantly larger average protein–protein interaction degrees than the background. Comparing the different cell types, the expression of four types of key genes showed specificity in different dimensions. Together, our results offer new insights into potential risk factors and help us understand the complexity and regional heterogeneity of SCZ.

## 1. Introduction

Schizophrenia (SCZ) is a severe chronic psychiatric disorder with a prevalence of <1% [1] and a heritability of 0.8 to 0.85 [2]. SCZ is characterized by positive symptoms, including delusions, hallucinations, and abnormal behavior, as well as negative symptoms, including social dysfunction, lack of motivation, and disorganized speech [3,4]. However, the genetic basis of SCZ remains largely undetermined, and the associations between SCZ’s clinical manifestation and genetics are unclear. Thus, identifying a potential genetic basis for SCZ is needed to understand how these complex components contribute to the disorder.

One of the primary goals of genomic medicine is to identify genetic risk factors for diseases. An important method used for discovering the genetic basis of diseases is based on using experimental mouse models to translate information from animals to humans. The abnormal behavior gene set (MP:0004924) derived from the Mouse Genome Informatics (MGI) database [5] is defined as any anomaly in the actions, reactions, or performance of an organism in response to external or internal stimuli compared to the controls. It is still a challenge to elucidate SCZ genes based on the evidence indicated by the abnormal behavior (AB) gene set.

To our knowledge, limited research has been carried out to systematically investigate which genes in the AB gene set are truly associated with SCZ. Today, the incremental growth of genetic studies provides another chance for using multi-omics integrative analysis to understand the etiology of SCZ, which has already resulted in hundreds of genetic loci being associated with SCZ [6]. Meanwhile, the links between psychiatric disorders and mutations, including copy number variants (CNVs) and *de novo* mutations (DNMs), have also been firmly established [7,8]. The Schizophrenia Exome Sequencing Meta-analysis (SCHEMA) consortium made an extensive effort to identify 10 exome-wide significant genes, with 24,248 cases and 97,322 controls [9]. Since the 22q11.2 deletion syndrome was first linked with SCZ [10], the research on SCZ and associated mutations has rapidly progressed. So far, the evidence for the roles of CNVs and DNMs in SCZ is overwhelming [11,12,13,14,15], as summarized in mutation databases such as PsyMuKB [16]. In addition, numerous SCZ transcriptome studies have identified various differentially expressed genes (DEGs) between patients and healthy controls [17,18], especially in brain regions such as the hippocampus (HIPPO) [17] and dorsolateral prefrontal cortex (DLPFC) [18,19]. However, determining the biological significance behind different brain regions is still a challenging task for SCZ research. In summary, both types of identified genes—genes with genetic variants (MUTs) and those with differentially expressed genes—may have different contributions to SCZ and may involve convergent or divergent biological functions. Analysis using the two types of gene sets could serve as a viable investigation method for identifying potential disorder risk factors based on the AB genes.

**Figure 1:**
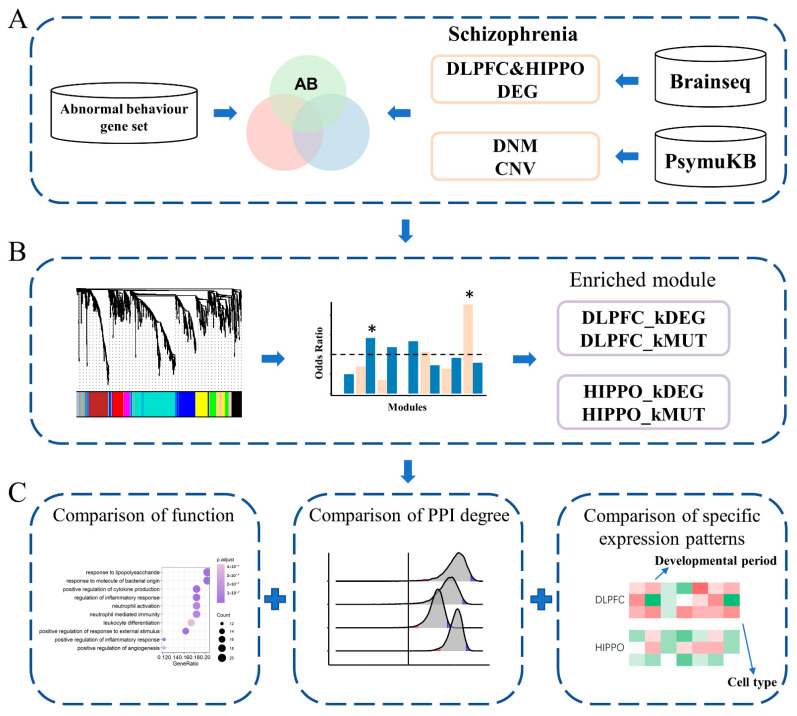
Flowchart of the study. (**A**) Data collection and filtration. RNA-Seq data analyzed in this study were obtained from BrainSeq Phase 2, which was also used to identify differentially expressed genes (DEGs). Copy number variants (CNVs) and *de novo* mutations (DNMs) associated with schizophrenia (SCZ) and the abnormal behavior (AB) gene set were collected from PsymuKB. DEGs and MUTs separately overlapped with the AB set. (**B**) The two co-expression networks were constructed from the AB gene expression profiles of two different brain regions separately (DLPFC and HIPPO). A star * with a significant *p*-value marks the identified module. (**C**) The integrative analyses pipeline, including protein–protein interaction (PPI) analysis, brain function, spatial-specific and cell-type-specific expression patterns, was followed. DLPFC, dorsolateral prefrontal cortex; HIPPO, hippocampus.

To better understand (1) which AB genes are the key genes of SCZ, (2) how the disruptions of these key genes in different brain regions are involved in SCZ, and (3) whether DEGs and MUTs are involved in convergent or divergent biological functions, we performed an integrative analysis (Figure 1). Two types of risk genes (DEGs and MUTs) were separately overlapped with AB genes from the PsyMuKB database. Then, we constructed the co-expression networks separately in DLPFC and HIPPO using the RNA-Seq expression data from BrainSeq Phase 2 [17]. Consequently, we identified four types of key genes affected by different types (DEGs and MUTs) in different brain regions (DLPFC and HIPPO): DLPFC-kDEG, DLPFC-kMUT, HIPPO-kDEG, and HIPPO-kMUT. Four types of key genes from the AB gene set were identified for subsequent analysis, including brain function, the protein–protein interaction (PPI) network, and spatial and cell-type-specific expression patterns, to uncover all possible underlying genetic links. Together, our results indicate that the key genes found by our integrative analysis could help in understanding and studying the potential risk genes in SCZ.

## 2. Materials and Methods 

### 2.1. Data Collection and Filtration

The RNA-Seq data analyzed in this study were obtained from BrainSeq Phase 2 [17], downloaded from http://eqtl.brainseq.org/phase2 (accessed on 17 December 2020) and consisting of 286 SCZ cases and 614 control samples. We used all 284 SCZ cases and 460 control samples over 18 years of age for subsequent analysis. Then, we chose genes that were expressed in the brain (defined as the average RPKM > 0.1 in BrainSeq Phase 2 [17]) for further analysis. The AB gene set (Table S1), CNV (Table S2), and DNM (Table S3) data for SCZ were collected from the PsyMuKB [16] in-house database (URL: http://www.psymukb.net/, accessed on 17 December 2020). AB genes with an average RPKM < 0.1 in BrainSeq were also removed.

### 2.2. Detection of Differentially Expressed Genes

Differential expression was assessed using the DESeq2 [20] package in R between SCZ cases and control samples separately by tissue from 2 brain regions: HIPPO and DLPFC. The significance threshold was an adjusted *p*-value of <0.05 and a log_2_ fold change (FC) value of >1.2.

### 2.3. Weighted Co-Expression Network Construction and Key Module Identification

The weighted co-expression network was built using the WGCNA [21] package in R. The networks were constructed separately by tissues, and AB genes with evidence of robust expression (see above) were included in the network. Then, network construction was performed using the blockwiseModules function. The soft-thresholding power was chosen based on the smallest threshold that resulted in a scale-free R2 fit of 0.9. We also set the minimum module size to 30 genes and the minimum height for merging modules to 0.25. Key modules were identified by the odds ratio. The module of each tissue with the highest odds ratio and *p*-value of <0.01 (Chi-square test) was chosen as the significant module for subsequent analysis.

### 2.4. Gene Ontology and Gene Set Enrichment Analyses

Unless otherwise noted, we used the enrichGO function from clusterProfiler [22] for gene ontology [23] enrichment analyses with a pvalueCutoff of 0.01 and a qvalueCutoff of 0.05. The gene-concept network was plotted using the cnetplot function of clusterProfiler.

### 2.5. Analysis of Spatial/Cell-type Specific Expression of Genes

We applied the Expression Weighted Celltype Enrichment (EWCE) R package [24], a bootstrap enrichment tool based on the specificity matrix, to conduct an enrichment analysis on two datasets: Brainspan [25] for the period and region analyses and Dronc [26] data for the adult brain cell type analysis. Specificity used the definition described by Saunders et al. (2018) [27]. We first calculated the specificity matrix of 2 single-cell datasets with the “generate.celltype.data” R function. Next, enrichment of all 4 types of genes was tested with the “bootstrap.enrichment.test” R function. The background gene list was defined by all genes annotated in the tested single-cell dataset.

### 2.6. Brain-Expressed PPI Statistics for Disease Genes

We calculated the numbers of PPI partners with other brain-expressed genes (bPPI) in the BioGRID [28] database for all brain-expressed genes. We then compared the average number of PPI partners for each gene type with other brain-expressed genes referred to as the background by bootstrapping 10,000 times. *p*-values were calculated using a 2-tailed hypothesis test. For example, DLPFC-kDEG contained 99 genes with an average of 35.93 PPI partners. We randomly chose 99 genes from among all the brain-expressed genes and calculated their average number of partners 10,000 times. In 9962 bootstraps, the average number was smaller than 35.93. Since the average number was larger than 35.93 in only 38 bootstraps, the *p*-value was 0.0038 for the alternative hypothesis “larger.”

## 3. Results

### 3.1. Abnormal Behavior Genes Were Significantly Enriched in SCZ Gene Sets

**Figure 2:**
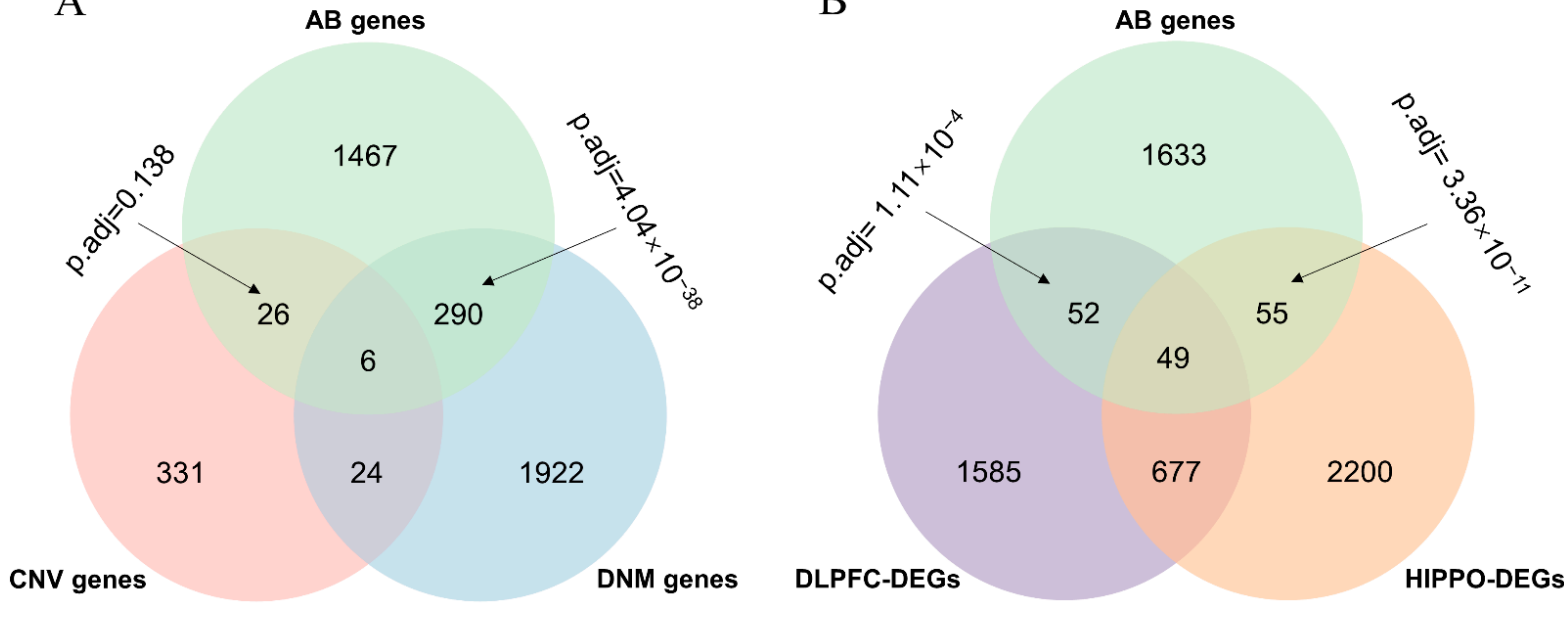
Overlaps between the AB gene set and different types of SCZ risk genes. (**A**) Venn diagram of AB and MUT by different types (CNV and DNM). (**B**) Venn diagram of AB and DEGs by brain region (DLPFC and HIPPO). The *p*-value was calculated by a hypergeometric test and adjusted by the Bonferroni method. AB genes, abnormal behavior gene set; CNV genes, genes associated with copy number variation; DNM genes, genes associated with *de novo* mutation; DLPFC-DEGs, differentially expressed genes detected in dorsolateral prefrontal cortex; HIPPO-DEGs, differentially expressed genes detected in hippocampus.

We focused on genes harboring mutations and differentially expressed genes in SCZ patients’ brains to define the subsets of different kinds of functional genes in SCZ. We first collected genes with genetic variants (MUTs), including CNVs and the DNMs of SCZ from PsyMuKB [16], as well as 2363 and 2981 genes that were identified to be differentially expressed between an adult with schizophrenia and a healthy control in DLPFC and HIPPO, respectively, with p.adj <0.05 and fold changes >1.2. Next, we extracted the AB gene set from PsyMuKB [16]. We observed a significant overlap between the AB gene set and the DNM gene (p.adj = 4.04 × 10^−38^ calculated using a hypergeometric test and adjusted with a Bonferroni test) but no significant overlap between the AB gene set and the CNV gene (p.adj = 0.138 calculated by a hypergeometric test and adjusted by a Bonferroni test) (Figure 2A). For DEGs, significant results were found for both overlaps between the AB gene set and DEGs in HIPPO (p.adj = 3.36 × 10^−11^ calculated by a hypergeometric test and adjusted by a Bonferroni test) and DLPFC (p.adj = 1.11 × 10^−4^ calculated by a hypergeometric test and adjusted by a Bonferroni test) (Figure 2B). In summary, except for the overlap between the AB gene set and the CNV gene, the other three overlaps were all significant.

### 3.2. Two Significant Modules Were Identified in DLPFC

**Figure 3:**
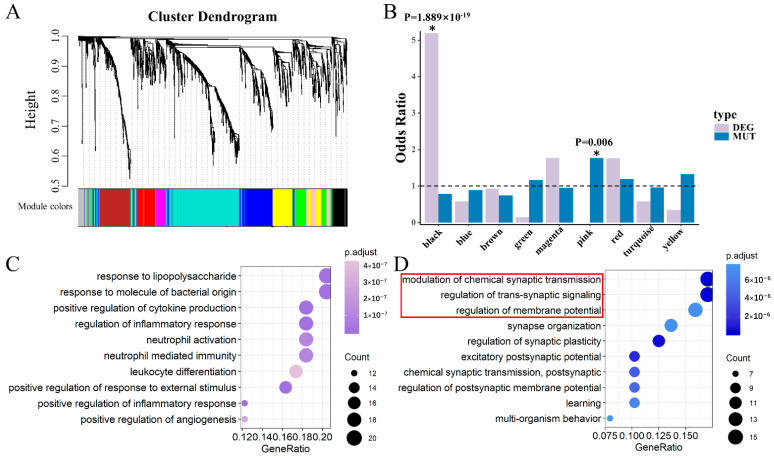
Module identification and functional enrichment in DLPFC. (**A**) Clustering dendrograms of the AB gene set according to gene expression data in DLPFC, with dissimilarity based on the topological overlap. Each colored row represents a color-coded module that contains a group of highly connected genes. In total, 10 modules were identified. (**B**) Modules are listed on the *x*-axis, and the *y*-axis indicates the odds ratio. The horizontal line indicates the threshold of y = 1, which distinguishes the modules enriched in a greater proportion by the target genes. The *p*-value was calculated by a Chi-square distribution test. A star with a significant *p*-value marks two identified modules. (**C**) GO-BP analysis for the black module genes (DLPFC-kDEG) showing the top 10 enriched gene ontology categories. Pathway names are shown on the left, and the color of dots on the right represents the adjusted *p*-value of the corresponding pathway. (**D**) Gene Ontology–Biological Pathway (GO-BP) analysis for pink module genes (DLPFC-kMUT) showing the top 10 enriched gene ontology categories. The red box indicates the GO terms that were the same as those in the blue module in HIPPO.

We performed a weighted gene co-expression network analysis (WGCNA) [21] to extract the biological information embedded in the multidimensional transcriptome dataset, which allowed us to identify modules of co-expressed genes. To identify discrete groups of co-expressed genes showing transcriptional differences between SCZ and the controls, we constructed a co-expression network using RNA-Seq data composed of both SCZ and control samples. Using a combination of SCZ cases (N = 152) and controls (N = 222) with the RNA-Seq expression data in the DLPFC from BrainSeq Phase 2 [17], 10 modules were identified, which were first examined for enrichment of DEGs and MUTs (Figure 3A). We found that DEGs overlapping with the AB gene set were significantly enriched in the black module (odds ratio (OR) = 5.189, Chi-square test *p* = 1.889 × 10^−19^) (Figure 3B). The MUTs of SCZ overlapped with the AB gene set and were significantly enriched in the pink module (OR = 1.768, *p* = 0.006) (Figure 3B). No significance was observed for any of the other modules. Thus, we defined genes within the black module as the keys of differentially expressed genes in DLPFC (DLPFC-kDEG) Likewise, genes within the pink module were considered the key genes affected by mutations in DLPFC (DLPFC-kMUT).

We next explored whether DLPFC-kDEG and DLPFC-kMUT diverged in their biological or neuronal functions. We performed Gene Ontology–Biological Pathway (GO-BP) analysis on DLPFC-kDEG and DLPFC-kMUT alone using the clusterProfiler [22] R package. DLPFC-kDEG was significantly enriched with gene ontology terms related to its response to lipopolysaccharides, positive regulation of cytokine production, and inflammatory response, among other factors. (Figure 3C). It was reported that stimulation of the peripheral blood mononuclear cells by lipopolysaccharides leads to the release of cytokines and other inflammatory mediators [29]. Schizophrenic patients featured an inflammatory cytokine profile and regulatory cytokines. The severity of symptoms may affect abnormal cytokine levels in these patients [30]. DLPFC-kMUT, on the other hand, was enriched in gene ontology categories related to the synapses, including modulation of chemical synaptic transmission, regulation of trans-synaptic signaling, and synapse organization (Figure 3D). We also found that 28 genes of DLPFC-kMUT overlapped with a synaptic gene group associated with SCZ, as identified by Lips et al. [31].

### 3.3. Two Significant Modules Were Identified in HIPPO

**Figure 4:**
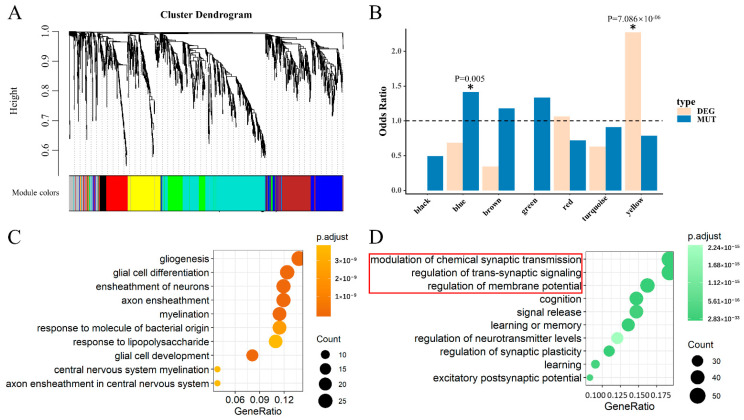
Module identification and functional enrichment in HIPPO. (**A**) Clustering dendrograms of the AB gene set by gene expression data in HIPPO with dissimilarity based on the topological overlap. A total of eight modules were identified. (**B**) The histogram shows significant modules. A star with a significant *p*-value marks two identified modules. (**C**) GO-BP analysis for yellow module genes (HIPPO-kDEG) showing the top 10 enriched gene ontology categories. (**D**) GO-BP analysis for blue module genes (HIPPO-kMUT) showing the top 10 enriched gene ontology categories. The red box indicates the GO terms that were same as those in the pink module in DLPFC.

To identify the key genes in HIPPO, we also constructed a co-expression network using similar analyses in DLPFC. With a combination of SCZ cases (N = 132) and controls (N = 238) using RNA-Seq data in the HIPPO from BrainSeq Phase 2 [17], eight modules were identified (Figure 4A). The yellow module was enriched in genes identified as DEGs overlapping with the AB gene set between the SCZ cases and controls, reflected by a significant odds ratio (OR = 2.272, *p* = 7.086 × 10^−6^) (Figure 4B). For the MUTs of SCZ overlapping with the AB gene set, the blue module was identified to be significantly enriched (OR = 1.415, *p* = 0.005) (Figure 4B). Genes of the yellow module were defined as the key for differentially expressed genes in HIPPO (HIPPO-kDEG). Likewise, the blue module genes were defined as the key genes affected by mutation in HIPPO (HIPPO-kMUT).

To determine whether there is a difference in biological or neuronal functions, we also performed a GO-BP analysis on HIPPO-kDEG and HIPPO-kMUT alone. We observed that the enrichment of HIPPO-kDEG was associated with gene ontology terms related to gliogenesis, glial cell differentiation, and the ensheathment of neurons, among other factors (Figure 4C). As observed, the top three gene ontology categories of HIPPO-kMUT were consistent with those of DLPFC-kMUT (Figure 4D). This result indicates that the MUTs of SCZ featured unified biological functions in both DLPFC and HIPPO based on the modulation of chemical synaptic transmission, the regulation of trans-synaptic signaling, and the regulation of membrane potential. Many studies have noted that subjects with SCZ appear to share a common abnormality in synaptic transmission control [32].

### 3.4. Comparison between DLPFC-kMUT and HIPPO-kMUT

**Figure 5:**
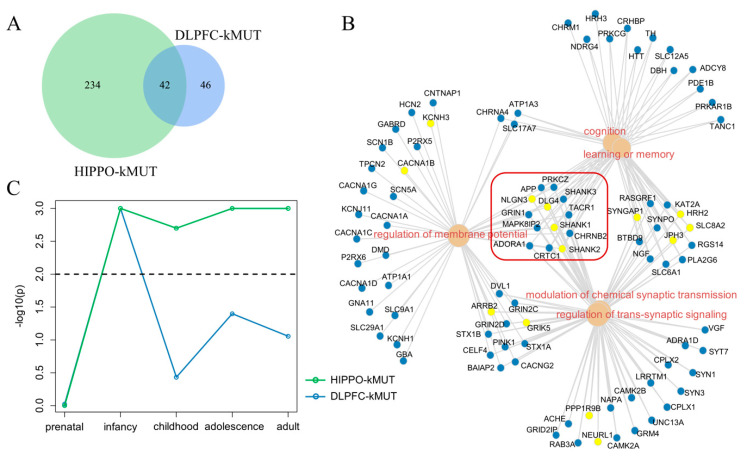
Comparison between DLPFC-kMUT and HIPPO-kMUT. (**A**) Overlapping gene counts of DLPFC-kMUT and HIPPO-kMUT. (**B**) GO-BP analysis for a combination of DLPFC-kMUT and HIPPO-kMUT, showing the genes contained in the first five most enriched GO terms. Blue dots show the genes contained in one of DLPFC-kMUT and HIPPO-kMUT, while the yellow dots show genes contained in both categories. The red box refers to genes related to all five GO terms. (**C**) Period-specific expression of DLPFC-kMUT and HIPPO-kMUT. The *p*-values calculated by the Expression Weighted Celltype Enrichment (EWCE) R package are shown. The *x*-axis shows the developmental stage, the *y*-axis shows the -log10 value of the p threshold, and the dashed line shows *p*-value = 0.01.

Considering the consistency of the top 3 GO terms of DLPFC-kMUT and HIPPO-kMUT, we compared the genes of the 2 categories and found that 42 genes were overlapped. Moreover, the overlap ratio of genes accounted for 15.2% of HIPPO-kMUT and reached 47.7% of DLPFC-kMUT (Figure 5A). 

Considering that DLPFC-kMUT and HIPPO-kMUT converged in their biological functions, we combined these two categories of genes to perform the functional enrichment analysis. As shown in Figure 5B, the five most significant GO terms were the modulation of chemical synaptic transmission (p.adj = 5.93 × 10^−33^), the regulation of trans-synaptic signaling (p.adj = 5.93 × 10^−33^), cognition (p.adj = 5.87 × 10^−23^), regulation of membrane potential (p.adj = 2.01 × 10^−22^), and learning or memory (p.adj = 2.84 × 10^−24^). The two terms (modulation of chemical synaptic transmission and regulation of trans-synaptic signaling) shared the exact same genes. In the same way, cognition and learning or memory nearly shared the same genes. We observed that 13 genes connected these 5 GO terms, 4 of which (SHANK1, SHANK2, DLG4, and NLGN3) belonged to the overlap between DLPFC-kMUT and HIPPO-kMUT.

After determining that DLPFC-kMUT and HIPPO-kMUT converged in GO enrichment, we sought to determine whether they also had specific expression patterns in terms of developmental periods. For this purpose, we applied the Expression Weighted Celltype Enrichment (EWCE) R package [24], a bootstrap enrichment tool based on a specificity matrix, to the Brainspan [25] dataset. The result showed similar trends during the early stages of development (prenatal and infancy) (Figure 5C). However, in childhood, adolescence, and adulthood, DLPFC-kMUT and HIPPO-kMUT diverged in the following trend: HIPPO-kMUT was specifically expressed in the child, adolescent, and adult brains, unlike that of DLPFC-kMUT. Although SCZ usually emerges between ages 18 and 25, several longitudinal population-based studies have indicated that problems appear much earlier [33].

### 3.5. Larger Average PPI Degree Compared with Background

**Figure 6:**
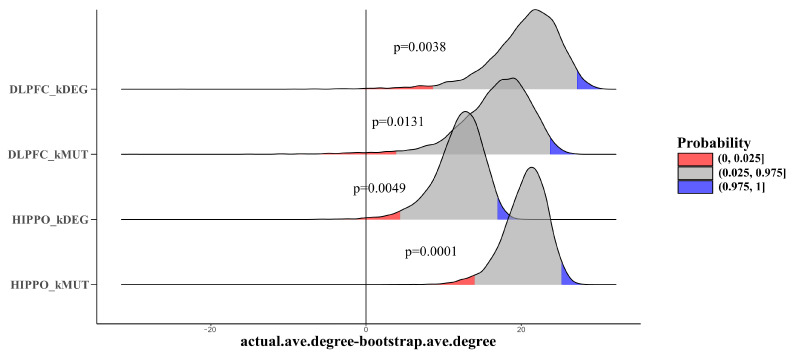
Joy plot shows bootstrap results comparing the average degrees of different kinds of key genes with other genes that are expressed in the brain (average RPKM > 0.1 in BrainSeq Phase 2). *x*-axis: The difference between the average degree obtained in the bootstrap tests and the actual average degree of the disease genes. *y*-axis: Density distribution. The horizontal line indicates x = 0, which means that the actual average degree equals the average bootstrap degree. The red and blue areas indicate the two-tailed test *p*-value threshold (*p* < 0.025 and *p* > 0.975).

Furthermore, we explored the roles of our key genes in the gene interaction networks. Protein–protein interactions are a key factor in determining protein function and are a basic component of cellular protein complexes and pathways since proteins rarely act alone. Comprehending PPI is thus crucial to understanding complex molecular relationships. To understand PPI characteristics, we used a bootstrap test (10,000 times) to compare the average degrees of four types of key genes with backgrounds defined by other genes that are expressed in the brain (average RPKM > 0.1 in BrainSeq Phase 2). Four types of key genes all showed a significantly larger average degree than the background (DLPFC-kDEG: *p* = 0.0344; DLPFC-kMUT: *p* = 0.0131; HIPPO-kDEG: *p* = 0.0049; HIPPO-kMUT: *p* = 0.0001) (Figure 6). In DLPFC-kDEG, for example, the P-value of the alternative hypothesis “smaller than the background” was 0.038, indicating that all four types of genes might act as hub genes in the PPI network.

### 3.6. Different Cell-Type-Specific Expression Patterns

We also tested the temporally specific expression patterns between DLPFC-kMUT and HIPPO-kMUT. As a result, we explored whether DLPFC-kMUT and HIPPO-kMUT have specific expression patterns in different cell types. We extended this comparison to all four categories via EWCE.

We also tested the brain cell-type-specific expression in Dronc single-cell data [26]. Comparing the different cell types, the expression of four categories of key genes showed specificity in different dimensions. On the one hand, DLPFC-kMUT and HIPPO-kMUT were specifically expressed in neuronal stem cells (NSC), microglia (MG), oligodendrocyte precursor cells (OPC), granule neurons from the hip dentate gyrus region (exDG), and GABAergic interneurons (GABA), while DLPFC-kDEG and HIPPO-kDEG were not specifically expressed. Conversely, DLPFC-kDEG and HIPPO-kDEG were specifically expressed in pyramidal neurons from the hip CA region (exCA1/3) (Table 1). Therefore, the gene types explained cell-type-specific expression patterns better than brain regions.

## 4. Discussion

In this study, we identified four categories of key genes (DLPFC-kDEG, DLPFC-kMUT, HIPPO-kDEG, and HIPPO-kMUT) in the AB gene set that may be potential SCZ risk factors and analyzed their characteristics. To our knowledge, this study is the first to focus on narrowing the scope of the AB mice gene set to determine the potential SCZ risk factors. These different categories of key genes will provide insights into the functional biology in SCZ.

In the analysis of gene co-expression, we identified a correlation between the SCZ and the AB gene set that strongly depends on the gene type class. In each co-expression network, only one module was found for each type of gene. When considering the same types of genes associated with different brain regions, strong concordance was found. As shown by our analysis and the genetic studies of others [34], different brain regions may share a genetic basis, especially in the early development stages. A study of gene expression patterns in the DLPFC of subjects with SCZ revealed that the gene’s encoding proteins involved in presynaptic function regulation were the most consistently altered [35].

The discovery of functional pathways is essential for explaining the biological processes of diseases [36]. Determining the essential regulatory pathways that can affect the brain’s molecular structure or function and that can lead to mental illness is an active research topic. Our finding that DLPFC-kMUT and HIPPO-kMUT share precisely the same top three GO terms associated with synaptic functions is worth exploring further. A previous study confirmed that DNMs on SCZ patients are significantly enriched in genes related to synaptic functions [37]. Furthermore, genes shared by the top five GO terms are highly associated with SCZ, including SHANK1/2/3, NLGN3, and DLG4. For the development stage, specific expression patterns of DLPFC-kMUT and HIPPO-kMUT converged in the early stage but diverged in the later stages of the developmental periods, which confirmed the notion that “early neurodevelopmental" injury may cause SCZ.

Post-synaptic neuroglial protein (NLGN) is one of the most well-characterized synaptic cell adhesion molecules, promoting excitatory and inhibitory synapse formation. In humans, there are five NLGN genes with NLGN1/2 located on the autosomes and NLGN3/4/4Y on the sex chromosomes. Hamilton et al. [38] assessed an Nlgn3 knockout mouse model that exhibited abnormalities in phenotypes, including juvenile play, perseverative behaviors, and sensorimotor gating. In a previous study, 19 genetic variants were identified by sequencing all the exons and promoter regions of the neuroligin-2 (NLGN2) gene with the cohort consisting of 584 SCZ patients and 549 control subjects, and the variant in NLGN2 was identified as a loss-of-function mutant in inducing GABAergic synaptogenesis, which may be an important contributing factor for the onset of SCZ [39]. The mRNA expression levels of NLGN3 and SHANK3 were found to be significantly decreased in individuals with autism spectrum disorder (ASD) compared to the controls [40]. SCZ and ASD are two severe psychiatric disorders that share considerable comorbidities in both clinical and genetic contexts [41]. The genetic influence of NLGN3 and SHANK3 on SCZ is also worth exploring.

Post-synaptic density protein 95 (PSD95) is a member of the synapse-associated protein family of scaffolding molecules that control the organization, composition, and function of synapses [42]. PSD95 is encoded by the disks large homolog 4 (DLG4) gene. Feyder et al. [43] characterized increased repetitive behaviors, abnormal communication and social behaviors, impaired motor coordination, and increased stress reactivity and anxiety-related responses in mice with PSD-95 deletion (Dlg4^−/−^). A family-based association analysis of genetic variants also highlighted a putative role for DLG4 in SCZ pathogenesis [44]. In addition, the linkage between DLG4 and SCZ has been well established through both variant association [45] and expression studies [46,47].

SHANK family members share five main domain regions: N-terminal ankyrin repeats, the SH3 domain, the PDZ domain, the proline-rich region, and a C-terminal SAM domain. Through these functional domains, SHANK interacts with many PSD proteins [48]. This complex has been shown to play an important role in targeting, anchoring, and dynamically regulating the synaptic localization of neurotransmitter receptors and signaling molecules [49]. Several genetic Shank mouse models have been generated, including Shank1 [50], Shank2 [51,52], and Shank3 [51,53,54,55] knockout mice models. In these studies, assays for detecting behavioral phenotypes in the following domains were included: (I) Social behavior, (II) communication, and (III) repetitive and stereotyped patterns of behavior. Numerous studies have strongly suggested a causative role of rare SHANK1/2/3 variants in SCZ [39,56,57,58] and have underlined the contribution of these variants in a variety of neuropsychiatric disorders. The genetic influence of SHANK1/2/3, NLGN3, and DLG4 on SCZ needs further experimental verification. In addition, the PSD95/SAPAP/SHANK postsynaptic complex may play an important role in SCZ. This is also worth exploring in subsequent research. SCZ and ASD are two different severe neurodevelopmental disorders with similar phenotypes and high comorbidity. It will be of great significance to study the common pathological mechanisms of the two disorders.

The four types of key genes all showed significantly larger average PPI degrees than the background. The availability of high-throughput PPI datasets has also led to various studies [59,60]. Previous analyses have suggested that an interactive network usually consists of a small number of highly connected “hubs” and many low degree nodes [61]. At the molecular level, highly connected “hub” genes are more sensitive to perturbations. Today, high-degree proteins remain a research hotspot. Our analysis suggests that these key genes involved in the abnormal behavior of experimental mouse models are more likely to be highly connected “hubs” in the network of SCZ.

Through macroscopic research, many remarkable results have been obtained. However, scientists have also begun to focus on the subtle differences between individual cells from the same organ or tissue to determine cell heterogeneity, which plays a vital role in complex neuropsychiatric illnesses. When studying cell-type-specific expression, DLPFC-kMUT and HIPPO-kMUT were found to be specifically expressed in the cells associated with neurons (NSC, MG, GABA, etc.). In SCZ, GABA may play an important role in the pathophysiology of SCZ due to changes in the presynaptic and postsynaptic components of its neurotransmission [62]. Using biological annotations and brain gene expression, we showed that the mutation class explains specific expression patterns better than specific brain regions.

In summary, although our current study has some limitations, we provided a strategy for discovering the potential genetic basis of diseases using experimental mouse models to translate information from animals to humans. Our strategy provides new insights into the possibility of studying potential risk genes of SCZ. These key genes we discovered may continue to be used to study mental disorders. These four categories of key genes provide new inspiration for follow-up experimental verification. These genes may serve as biomarkers to be applied as potential therapeutic targets for SCZ. Targets identified because they inhabit high-confidence networks related to both risk and the illness state may act better than specific gene candidates. These current findings on key genes likely foreshadow the regional heterogeneity and biological differences in the gene types of SCZ. The present integrative analysis strengthens our understanding of SCZ and enhances our ability to find new ways to improve the lives of individuals affected by this disorder.

## 5. Conclusions

In our study, we identified four categories of key genes in an abnormal behavior gene set that may be potential SCZ risk factors (DLPFC-kDEG, DLPFC-kMUT, HIPPO-kDEG, and HIPPO-kMUT) and analyzed their characteristics. We found a similar synaptic function between DLPFC-kMUT and HIPPO-kMUT. For the development stage, specific expression patterns of DLPFC-kMUT and HIPPO-kMUT converged in the early stage of development and diverged later. The four types of key genes all showed a significantly larger average PPI degree than the background. Through cell-type-specific expression patterns, gene types explained cell-type-specific expression patterns better than brain regions. These different categories of key genes may provide insight for selecting biomarkers of SCZ.

## Figures and Tables

**Table 1:**
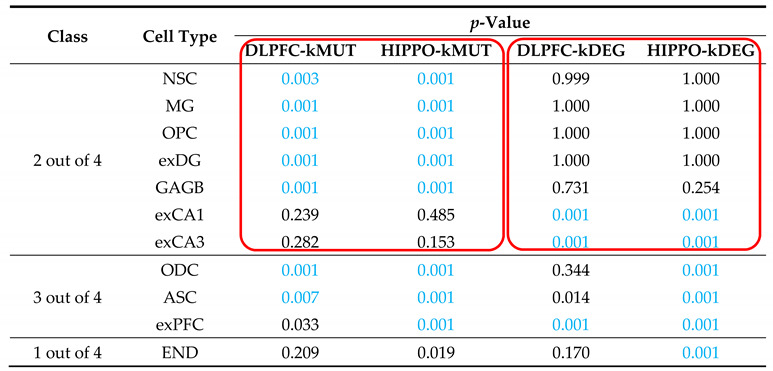
Cell-type expression patterns of DLPFC-kDEG, DLPFC-kMUT, HIPPO-kDEG, and DLPFC-kMUT. Cell-type-specific expression of four types of genes. The *p*-values calculated by the EWCE R package are shown. *p*-values < 0.01 are marked as blue. Classes are divided by the number of specific expressions in the four categories. The red boxes indicate categories with similar specific expression patterns. NSC, neuronal stem cell; MG, microglia; OPC, oligodendrocyte precursor cell; exDG, granule neuron from the hip dentate gyrus region; GABA, GABAergic interneuron; exCA1/3, pyramidal neuron from the hip CA region; ODC, oligodendrocyte; ASC, astrocyte; exPFC, glutamatergic neuron from the PFC; END, endotheliocyte.

## Supplementary Materials

The following are available online at https://www.mdpi.com/2075-1729/11/2/172/s1, Table S1: Abnormal behavior gene set used in this study, Table S2. Details of genes with copy number variant uesd in this study from PsyMuKB, Table S3. Details of genes with *de novo* mutation uesd in this study from PsyMuKB.
